# Electric field strength, hippocampal volume change, and cognitive outcomes following electroconvulsive therapy: the role of sex and anatomy

**DOI:** 10.1016/j.nicl.2026.104078

**Published:** 2026-10-09

**Authors:** Margherita Biondi, Eivind Haga Ronold, Ingrid Mossige, Leif Oltedal, Olga Therese Ousdal, Christopher C. Abbott, Ketil Joachim Ødegaard, Renate Grüner, Ute Kessler

**Affiliations:** a Padova Neuroscience Center, University of Padova, Via Giuseppe Orus, 2, 35131 Padova, Italy; b Department of Clinical and Biological Psychology, University of Bergen, Christies gate 12, 5015 Bergen, Norway; c Mohn Medical Imaging and Visualization Centre, Department of Radiology, Haukeland University Hospital, Haukelandsveien 22, 5009 Bergen, Norway; d Department of Physics and Technology, University of Bergen, Allégaten 55, 5007 Bergen, Norway; e Department of Physics and Computational Radiology, Division of Radiology and Nuclear Medicine, Oslo University Hospital, Sognsvannveien 21, 0372 Oslo, Norway; f Department of Clinical Medicine, University of Bergen, Haukelandsveien 28, 5009 Bergen, Norway; g Department of Biomedicine, Faculty of Medicine, University of Bergen, Jonas Lies vei 91, 5009 Bergen, Norway; h Department of Psychiatry, University of New Mexico, 2600 Marble Ave, Albuquerque, NM 87106, USA; i Department of Psychiatry, Haukeland University Hospital, Sandviksleitet 1, 5036 Bergen, Norway

**Keywords:** ECT, Depression, Electric field, Hippocampus, Cognition, Sex

## Abstract

**Background:**

Electroconvulsive therapy (ECT) induces hippocampal volume alterations, but the clinical significance of the volumetric change remains unclear. Greater electric field (EF) strength has been associated with increases in hippocampal volume and decline in cognitive performance following ECT, and this association might be moderated by individual-level variables, such as sex.

**Methods:**

In this study, 29 patients with severe or treatment-resistant depression underwent right unilateral ECT. EF strength was simulated using ROAST, and hippocampal volumes were segmented with FreeSurfer. Cognitive performance was assessed pre- and post-ECT using a multidomain neuropsychological battery. Associations between EF strength, hippocampal volume change, and cognitive outcomes were analyzed, with sex as a moderator.

**Results:**

At the group level, ECT reduced depressive symptom severity, without cognitive decline. Female patients exhibited higher EF strength in bilateral hippocampi, associated with smaller intracranial volumes and greater changes in the left hippocampal volume following ECT. Exploratory analyses indicated that higher EF strength and greater left hippocampal volume increase were linked to smaller improvement in verbal memory performance; moreover, a sex×volume interaction suggested opposite trends by sex, with volume increase correlating with greater improvement in males but smaller in females. Greater hippocampal volume change was also linked to lower consistency in autobiographical memory; however, *post-hoc* sensitivity analysis indicated that this association was outlier-driven and should be interpreted with caution.

**Conclusions:**

Our findings suggest that individual anatomical variability influences the EF and may contribute to cognitive side effects following ECT. Awareness of anatomy-related EF differences may inform future research strategies for individualized treatment planning and cognitive monitoring.

## Highlights

• ECT reduced depressive symptoms without group-level cognitive decline.
• Smaller intracranial volume was linked to higher hippocampal electric fields.
• Higher left hippocampal EF exposure was linked to less verbal memory improvement.
• Sex-specific patterns in volume change and verbal memory were exploratory.
• Anatomy may be more relevant than sex per se to cognitive vulnerability in ECT.
• Unexpected memory difficulties should prompt review of the ECT protocol.

## 1 Introduction

Electroconvulsive therapy (ECT) is a well-established and highly efficacious treatment option mainly for patients with severe or treatment-resistant depression (TRD) (The UK ECT Review Group, 2003). ECT involves the application of a controlled amount of electrical current through electrodes to the scalp, inducing a seizure while the patient is under anesthesia. Although the seizure is considered necessary for the antidepressive effect, the precise mechanism remains unclear, but ECT is known to induce structural and functional alterations in the brain (Ousdal et al., 2022).

Increasing evidence suggests that changes in grey matter volume following ECT are widespread throughout various regions of the brain, with highest effect sizes in the hippocampus and amygdala (Gryglewski et al., 2021; Ousdal et al., 2020). A particular factor associated with these anatomical alterations is the strength of the electric field (EF), suggesting the electrical stimulus itself may play a central role in driving volume alterations (Argyelan et al., 2019; Deng et al., 2022). However, other physiological parameters, such as cumulative seizure duration, have also been implicated (Takamiya et al., 2021), indicating that the mechanisms underlying ECT-induced brain changes are multifactorial and not yet fully understood.

The hippocampus is involved in mood regulation, learning, and memory, and it is known to be volumetrically reduced in patients with depression compared to healthy controls (for the ENIGMA-Major Depressive Disorder Working Group et al., 2016). Neuroimaging studies show significant increases in hippocampal volume following ECT (Gryglewski et al., 2021; Nordanskog et al., 2010; Wilkinson et al., 2017), yet the clinical significance of these changes remains unclear (Wilkinson et al., 2017; Oltedal et al., 2018). ECT is associated with cognitive side effects, particularly in verbal and autobiographical memory (Landry et al., 2021; Mathiassen et al., 2025), and some of these side effects are related to volume increase of the hippocampus (Argyelan et al., 2021; Gbyl et al., 2021; Ousdal et al., 2025; Van Oostrom et al., 2018).

The distribution of the EF is determined by the configuration of the ECT electrodes and the patient's head anatomy, while the strength of the EF depends on the amplitude of the ECT stimulus current (Peterchev et al., 2010). Given the EF's association with structural changes in brain regions critical for cognitive function, it may play a role in both therapeutic and adverse cognitive effects of ECT, as suggested by several recent studies. Deng et al. (2022) found that higher EF strength in the right hippocampus was associated with poorer phonemic fluency, suggesting that increased EF may compromise cognitive safety (Deng et al., 2022). Qi et al. (2022) demonstrated that higher EF strength within a cognitive network was correlated with greater cognitive impairment in a sample of late-life ECT patients (Qi et al., 2022). Both Abbott et al. (2024) and Loef et al. (2025) showed that higher EF strength was associated with decline in verbal fluency, while no association was found between EF and antidepressant response (Abbott et al., 2024; Loef et al., 2025).

One potential moderator of these effects is biological sex. Some evidence suggests that females may be more susceptible to ECT-related cognitive side effects. For example, subjective memory worsening was more frequently reported by females in a registry study (Brus et al., 2017), and Sackeim et al. (2007) found that female sex, along with older age and lower scores on measures of intellectual functioning, predicted greater cognitive impairment following ECT (Sackeim et al., 2007). One contributing factor to this vulnerability to ECT-induced cognitive side effects may be the lower seizure threshold observed in females compared to males (Sackeim, 1987). Additionally, anatomical factors such as skull thickness, head diameter, and brain volume, which are largely sex-dependent, can impact the distribution of the EF and the subsequent volume changes (Peterchev et al., 2010). Investigating the anatomical variability (geometry and conductivity of the various tissues) and how this affects the spatial distribution of the EF in the brain can clarify whether sex moderates the relationship between EF strength, hippocampal volume changes, and cognitive outcomes and help refine treatment.

The current study investigated the impact of EF strength on hippocampal volume change and cognitive functioning in a sample of patients undergoing ECT due to severe depression or TRD, focusing on whether sex moderates these associations. We hypothesize that higher EF strength will be associated with greater hippocampal volume increase and poorer cognitive performance, and that these associations will be moderated by sex.

## 2 Methods and materials

The data in the current paper stem from a prospective observational study (Registered at ClinicalTrials.gov, identifier: NCT04348825) and build upon the thesis of Mossige (2021) (Mossige, 2021). Patients provided written informed consent prior to inclusion in the study. The study was approved by the Regional Committee for Research Ethics in South East Norway (2013/1032).

### 2.1 Participants and procedure

Patients receiving ECT for major depressive episodes (uni- and bipolar depressive disorder) at the Haukeland University Hospital in Bergen, Norway were recruited. The study protocol has been described previously (Oltedal et al., 2015). A total of 40 patients over 18 years old, with a Montgomery and Åsberg Depression Rating Scale (MADRS) score > 25 (Montgomery and Åsberg, 1979), not having received ECT within the last 12 months were included, and MRI scans pre- post treatment was available for 29.

### 2.2 ECT treatment

ECT was administered with a Thymatron System IV (Somatics, Lake Bluff, IL, USA), providing brief square pulses with a constant current of 900 mA. The stimulus was applied via right unilateral (RUL) electrode placement and with a pulse width of preferably 0.5 ms (range 0.25–1.00 ms). The initial stimulus dose was determined by an age-based method and could be adjusted if indicated. In order to consider sex-specific differences in seizure threshold, the dose was adapted as follows: for male patients: % Energy + 5 to 10%; for female patients: % Energy - 5 to 10%. Three sessions per week were given until remission or until no further improvement of symptoms was expected, with a maximum of 20 sessions. We recorded the number of treatments, the stimulus average dose and cumulative dose, and the exposure time (see Table 1).

**Table 1:** Patients' demographics, clinical characteristics, and ECT technical parameters.

	Patients (*n* = 29)	Females (*n* = 15)	Males (*n* = 14)	*t/W/χ*^*2*^ statistic Females vs Males	*p*-value
Age (years)	48±15	43±14	52±16	*t* = −1.52	0.14
IQ	108±15	102±16	114±13	*W* = 32	0.04
Education (years)	14±4	13±4	14.5±4	*t* = −0.76	0.46
Age at first symptom (years)	26±15	25±16	27±15	*W* = 96.5	0.73
BMI	25±4	24±4	26±4	*t* = −1.56	0.13
Diagnosis (%)	UP (76%)	UP (66%)	UP (86%)	*χ*^*2*^ = 0.18	0.67
	BP (24%)	BP (33%)	BP (14%)	*χ*^*2*^ = 1.29	0.26
Length of current depressive episode (weeks)	48±46	58±54	36±34	*W* = 115.5	0.24
Presence of psychotic symptoms (%)	Yes = 7%	Yes = 7%	Yes = 7%	*χ*^*2*^ = 0	1
	No = 93%	No = 93%	No = 93%	*χ*^*2*^ = 0.04	0.85
Medications (%)	AD (83%)	AD (73%)	AD (93%)	*χ*^*2*^ = 0.17	0.68
	AE (17%)	AE (20%)	AE (14%)	*χ*^*2*^ = 0.2	0.65
	AP (83%)	AP (87%)	AP (79%)	*χ*^*2*^ = 0.17	0.68
	BZD (21%)	BZD (13%)	BZD (29%)	*χ*^*2*^ = 0.67	0.41
	LI (21%)	LI (20%)	LI (21%)	*χ*^*2*^ = 0	1
Number of ECT sessions	10±4	10±4	10±4	*t* = −0.33	0.75
Average dose (mC)	249±110	204±63	298±129	*W* = 49	0.01
Cumulative dose (mC)	2528±1624	1987±1115	3108±1909	*W* = 74	0.19
Exposure time (s)	3±2	2±1	3.5±2	*W* = 74	0.19

Mean ± standard deviation of age, IQ, education (years), age at first symptom, BMI, length of current depressive episode (weeks), number of ECT sessions, average dose (mC), cumulative dose (mC), and exposure time (s) were reported. Abbreviations: AD = Antidepressants, AE = Antiepileptics, AP = Antipsychotics, BMI = Body Mass Index, BP = Bipolar depressive disorder, BZD = Benzodiazepines, ECT = Electroconvulsive Therapy, IQ = Intelligence Quotient, LI = Lithium, UP = Unipolar depressive disorder.

### 2.3 MRI acquisition

T1-weighted volumetric MRI was obtained twice (within 1–2 h pre-ECT; 7–14 days post-ECT). Scans were acquired at 3 T. Full scanner models, coil details, and sequence parameters are provided in Supplementary Methods (M1).

### 2.4 Image analysis and EF simulation

Hippocampal volumes were derived using FreeSurfer's longitudinal pipeline (Reuter et al., 2012), and automated subcortical segmentation (`aseg`). Hippocampal volumes were extracted from the standard FreeSurfer hippocampal labels in `aseg.stats`. FreeSurfer's automated subcortical segmentation is based on a probabilistic labeling approach described by Fischl et al. (2002) (Fischl et al., 2002). EF simulation in ROAST (Huang et al., 2019) was used to derive the EF strength. A representative example of the right unilateral electrode configuration and the resulting spatial distribution of the simulated EF is shown in Supplementary Fig. S1. See Supplementary Methods (M2) for processing details.

### 2.5 Clinical and cognitive assessment

The severity of depressive symptoms was assessed with the MADRS by the treating clinician before (t_1_) and after (t_2_) an ECT-series. Remission was defined as a MADRS score lower than 10 (Oltedal et al., 2015). Cognitive function was assessed at t_1_ and t_2_ with a multidomain neuropsychological battery (CVLT-II, REY, Verbal Fluency, AMI-SF, EMQ, WASI). Full tests description is provided in Supplementary Methods (M3).

### 2.6 Statistical analyses

Statistical analyses were performed using R Statistical Software, version 4.1.3 (R Core Team, 2022). Group differences were examined using *t*-tests, χ^2^ tests, or Mann-Whitney *U* tests as appropriate. Linear regression models were performed to analyze the relationship between the simulated EF strength in the hippocampi and explanatory variables such as sex, age, and intracranial volume; equivalent models assessed percentage hippocampal volume change. To address confounding factors, multivariable models included BMI, age, sex, number of ECT sessions, and intracranial volume. Associations between continuous variables were examined using Pearson's *r* correlation coefficients. Group differences by sex were examined using *t*-tests or Mann-Whitney *U* tests, depending on distributional assumptions.

Cognitive change was defined as Δ = t_2_ − t_1_. We additionally report within sample effect sizes (ES) as the mean Δ divided by the baseline SD (a clinical approximation to Cohen's *d*; thresholds ∼0.2/0.5/0.8 denote small/moderate/large) (Borland et al., n.d.). Linear regression models related change in cognitive scores to hippocampal volume change or to hippocampal EF exposure (i.e., mean EF × cumulative exposure time), adjusting for age, sex, and clinical change (ΔMADRS); IQ was additionally included for analyses of CVLT-II, REY, and Verbal Fluency. Number of ECT sessions was not included because it is embedded in the EF-exposure variable. To further investigate potential sex-related differences, sex moderation was tested via interaction terms (hippocampal volume change × sex and hippocampal EF exposure × sex).

Outliers were identified using the interquartile range (IQR) method applied to Δ cognitive scores, and sensitivity analyses repeated models without them. Given the exploratory design and limited power, we used two-sided α = 0.05 without corrections for multiple comparisons.

## 3 Results

### 3.1 Patient characteristics

The demographics and clinical characteristics for the 29 included patients as well as ECT technical parameters are listed in Table 1. Female and male patients differed in IQ (*W* = 32, *p* = 0.04) and ECT average dose (*W* = 49, *p* = 0.01).

### 3.2 Simulated electrical field strength in the hippocampus

Higher EF strengths were found in both the left (*t* = −4.13, *p* < 0.001) and the right (*t* = −4.79, *p* < 0.0001) hippocampi of female patients compared to male patients, as shown in Fig. 1A. Further, EF strength was negatively associated with age (left: *r* = −0.64, *p* = 0.0002; right: *r* = −0.50, *p* = 0.006). EF strength was also negatively associated with intracranial volume (left: *r* = −0.53, *p* = 0.003; right: *r* = −0.58, *p* < 0.001), see Fig. 1B. Intracranial volume differed significantly by sex, with female patients having smaller intracranial volumes than male patients (*t* = −5.23, *p* < 0.001). In multivariable models including sex, age, intracranial volume, and BMI, sex was no longer a significant predictor (left: *p* = 0.3; right: *p* ≈ 0.09), suggesting that head size (intracranial volume) accounts for most of the observed sex differences. Age remained significant for the left hippocampus (*β* = −19.9, *p* = 0.001), while intracranial volume also contributed (*β* = −13.7, *p* = 0.03).

**Fig. 1:**
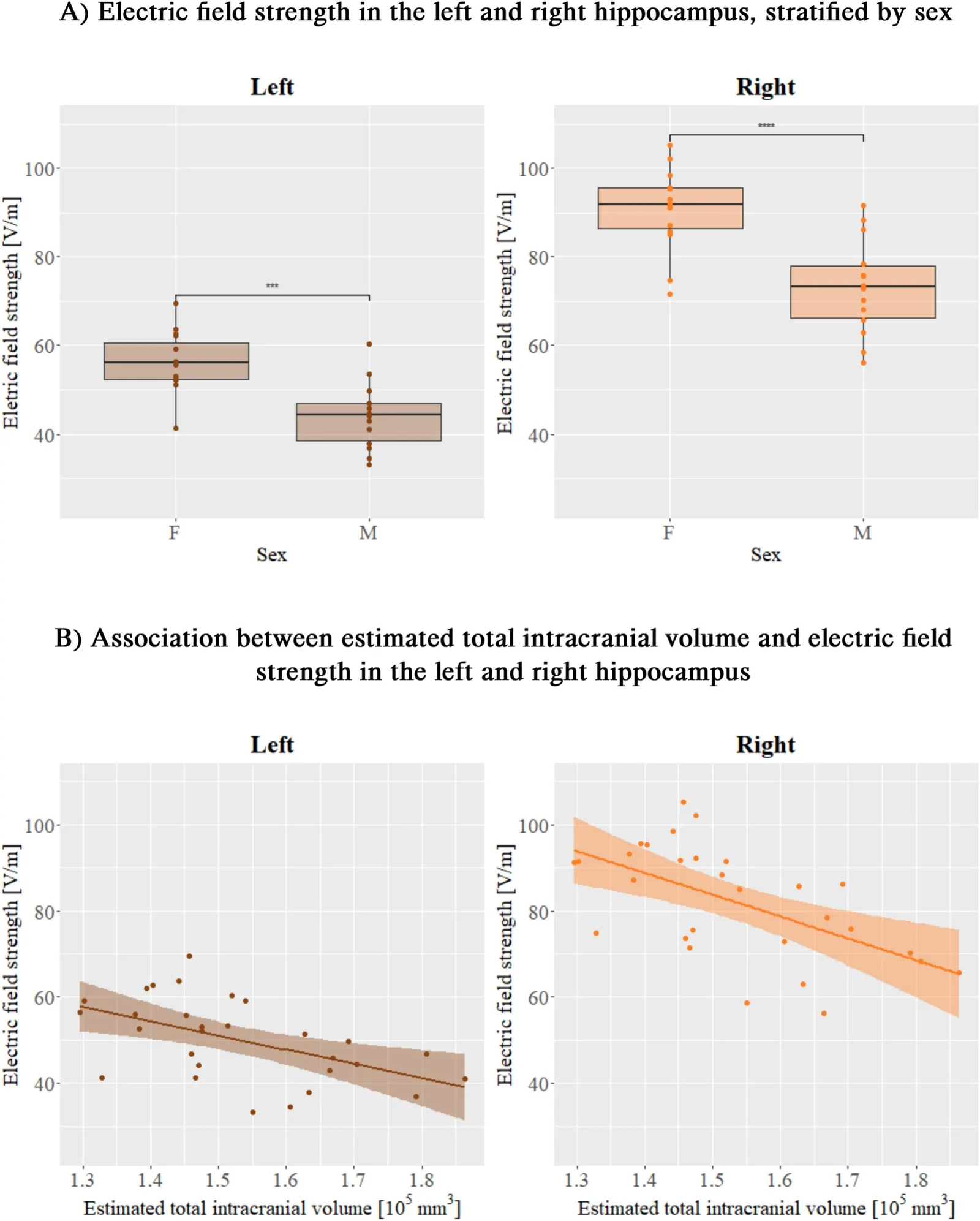
Electric field strength in the hippocampus by sex and intracranial volume. (A) There is a higher EF strength in both the left and right hippocampus in females compared to males. (B) Further, there is a strong negative association between EF strength and intracranial volume. Adapted from Mossige (2021) (Mossige, 2021).

### 3.3 Volume change of the hippocampus

Female patients exhibited greater left hippocampal volume change compared with males (*t* = −2.2, *p* = 0.04), with increases of approximately 3–4% versus 1%, respectively. In contrast, sex differences were not significant for the right hippocampus, see Fig. 2A. Analysis of percentage volume change revealed a positive association between EF strength and volume increase in the left hippocampus (*r* = 0.46, *p* = 0.01), whereas no relationship was observed on the right side, see Fig. 2B. The number of ECT sessions correlated positively with volume change in the right hippocampus (*r* = 0.46, *p* = 0.01) (Fig. S2). No associations were found between hippocampal volume change and age or BMI. Multiple linear regression models combining EF strength, age, sex, BMI, and number of ECT sessions did not reach overall significance for the left hippocampus (*p* = 0.06) or the right hippocampus (*p* = 0.09), although the number of ECT sessions remained a significant predictor for the right hippocampus (*β* = 0.047, *p* = 0.007). Finally, EF exposure (EF strength × total exposure time) showed no association with volume change in the left hippocampus, but was positively correlated with volume change in the right hippocampus (*r* = 0.44, *p* = 0.02).

**Fig. 2:**
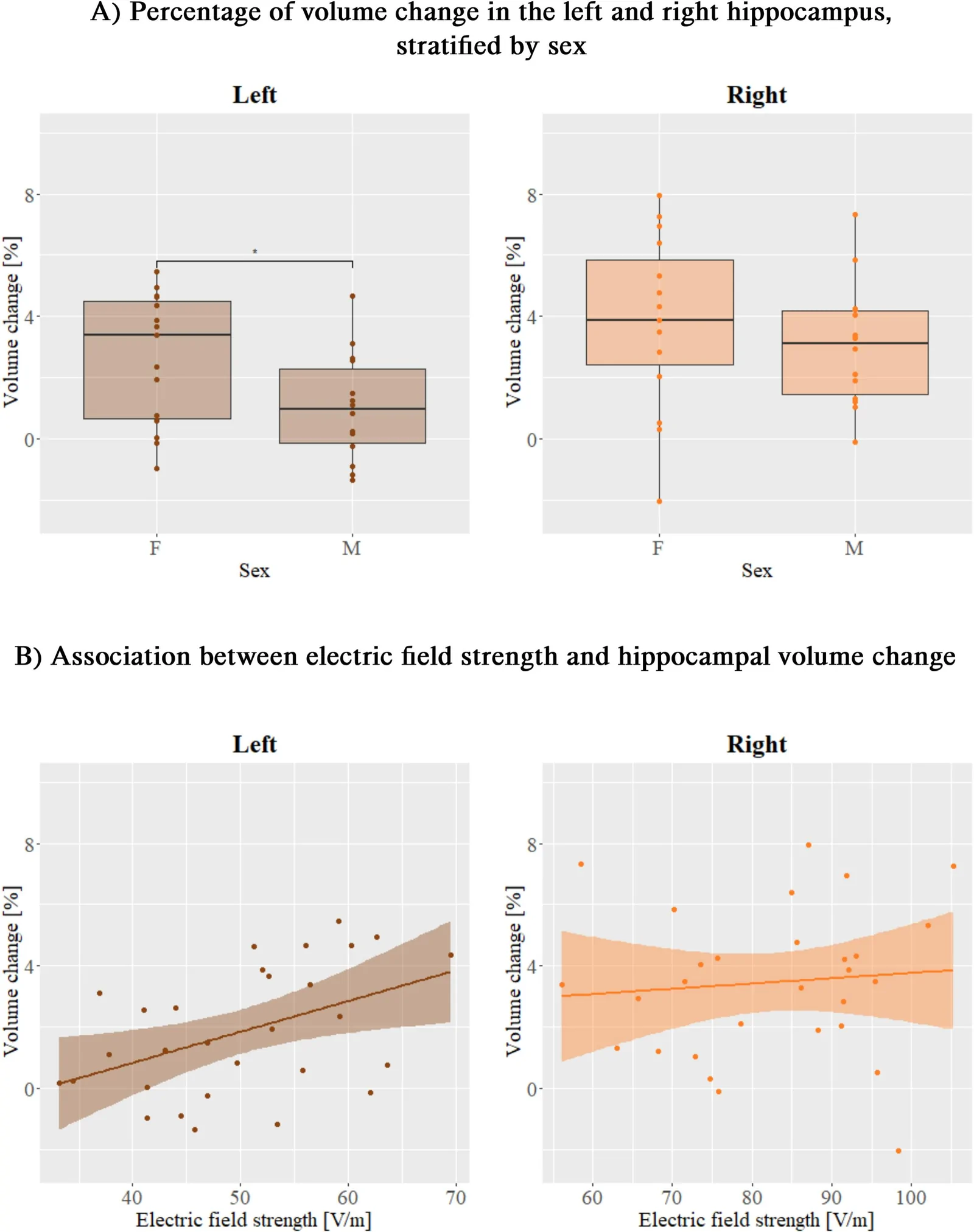
Hippocampal volume change by sex and electric field strength. (A) Female patients exhibited greater percentage volume change in the left hippocampus compared to males, while differences on the right side were minimal. (B) Further, there is a positive correlation between electric field strength and volume change in the left hippocampus, with no association observed for the right hippocampus. Previously presented in Mossige (2021) (Mossige, 2021).

### 3.4 Clinical and cognitive assessment

ECT patients had reduced MADRS scores after treatment (*t* = 11.01, *p* < 0.001) (Table S1). Mean cognitive test scores showed numerical increases (improvement) on all tests after ECT, but these changes were not statistically significant (see Supplementary Results, Table S1). Further, no sex difference in cognitive performance was observed, as no between-group difference in any of the cognitive tests emerged (see Supplementary Results, Table S2).

### 3.5 Change in cognitive performance and the association with hippocampal volume change

Exploratory analyses revealed sex-dependent patterns for CVLT-II and AMI-SF, with interaction effects suggesting opposite associations between hippocampal volume change and cognitive outcomes in males versus females.

CVLT-II (verbal memory): Increased left hippocampal volume was associated with smaller improvement in performance on CVLT-II overall (*b* *=* −2.21*, t* = −2.33, *p* = 0.04). A significant interaction with sex (*b* *=* 1.37, *t* = 2.15, *p* = 0.05) indicated that volume increase correlated with greater improvement in performance in males but smaller in females (Fig. 3A).

**Fig. 3:**
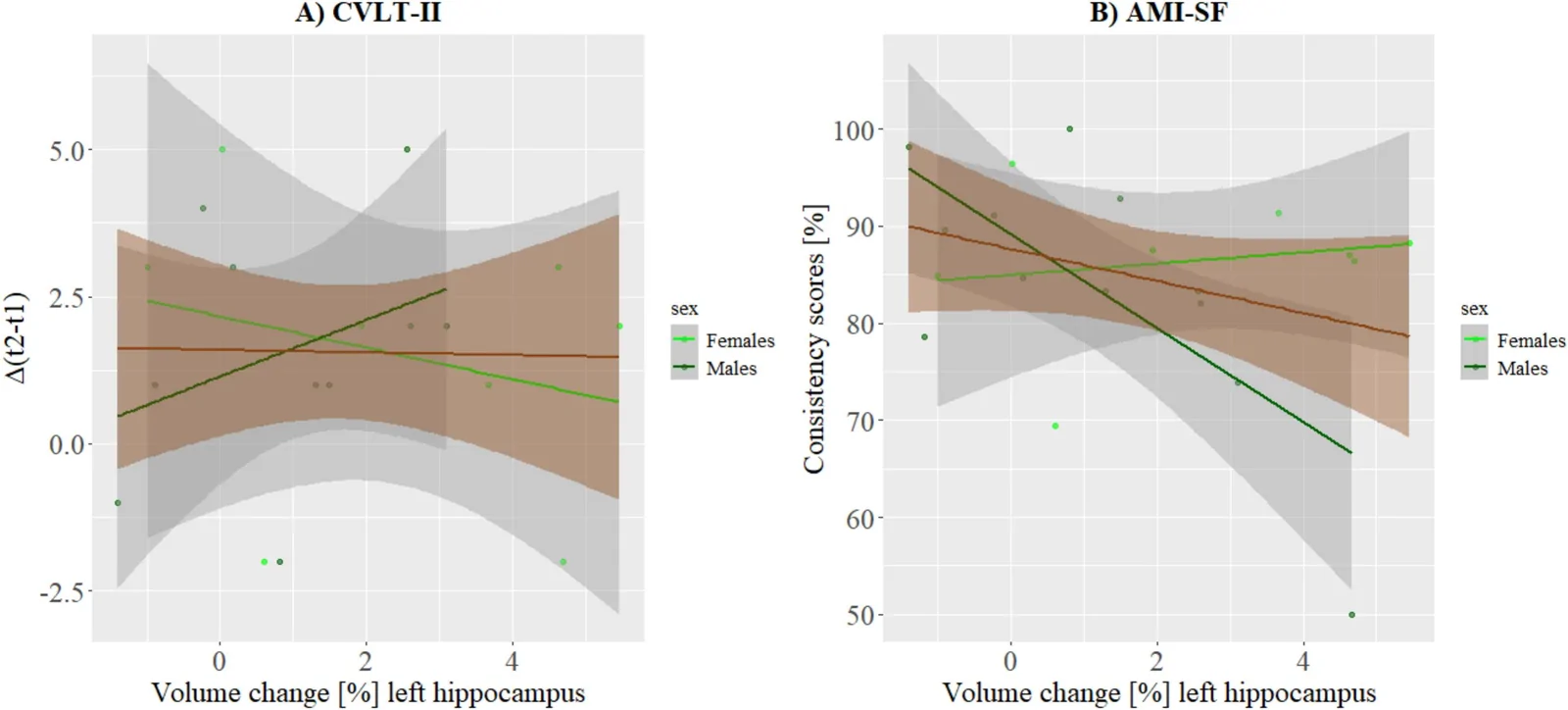
CVLT-II Δ scores and AMI-SF consistency scores associated with volume change in the left hippocampus and the interaction with sex. Relationship between left hippocampal volume change and (A) change in CVLT-II performance and (B) AMI-SF consistency scores, both stratified by sex. In panel A, positive Δ values (>0) are indicative of an improvement in performance on CVLT-II. Regression lines represent adjusted linear regression models controlling for age, sex, ΔMADRS, and IQ. Shaded areas indicate 95% confidence intervals. Abbreviations: AMI-SF = Autobiographical Memory Interview-Short Form, CVLT-II = California Verbal Learning Test-Second Edition.

AMI-SF (autobiographical memory): Lower consistency scores were linked to greater left hippocampal volume change (*b* *=* −4.87, *t* = −2.83, *p* = 0.01). The interaction with sex (*b* *=* 5.31, *t* = 2.29, *p* = 0.04) showed opposite trends: positive association in females and negative in males (Fig. 3B). *Post-hoc* sensitivity analysis indicated these results were influenced by outliers, which were retained to reflect natural variability in this heterogeneous sample.

No associations were found between changes in hippocampal volume and scores on other cognitive tests (see Supplementary Results, Table S3 and Table S4).

### 3.6 Change in cognitive performance and the association with EF exposure

Our final analysis tested whether higher EF exposure in the left and right hippocampus during ECT could predict differences in Δ cognitive scores.

Significant results were observed only for CVLT-II. The adjusted model demonstrated that higher EF exposure in the left hippocampus predicted smaller improvement in performance on CVLT-II (*b* *=* −0.02, *t* = −2.16, *p* = 0.05, Fig. 4). For the right hippocampus, the adjusted model indicated a trend toward significance in the same direction (*b* *=* −0.01, *t* = −2.03, *p* = 0.06). No associations were found between EF exposure in the hippocampus and scores on other cognitive tests (see Supplementary Results, Table S5 and Table S6).

**Fig. 4:**
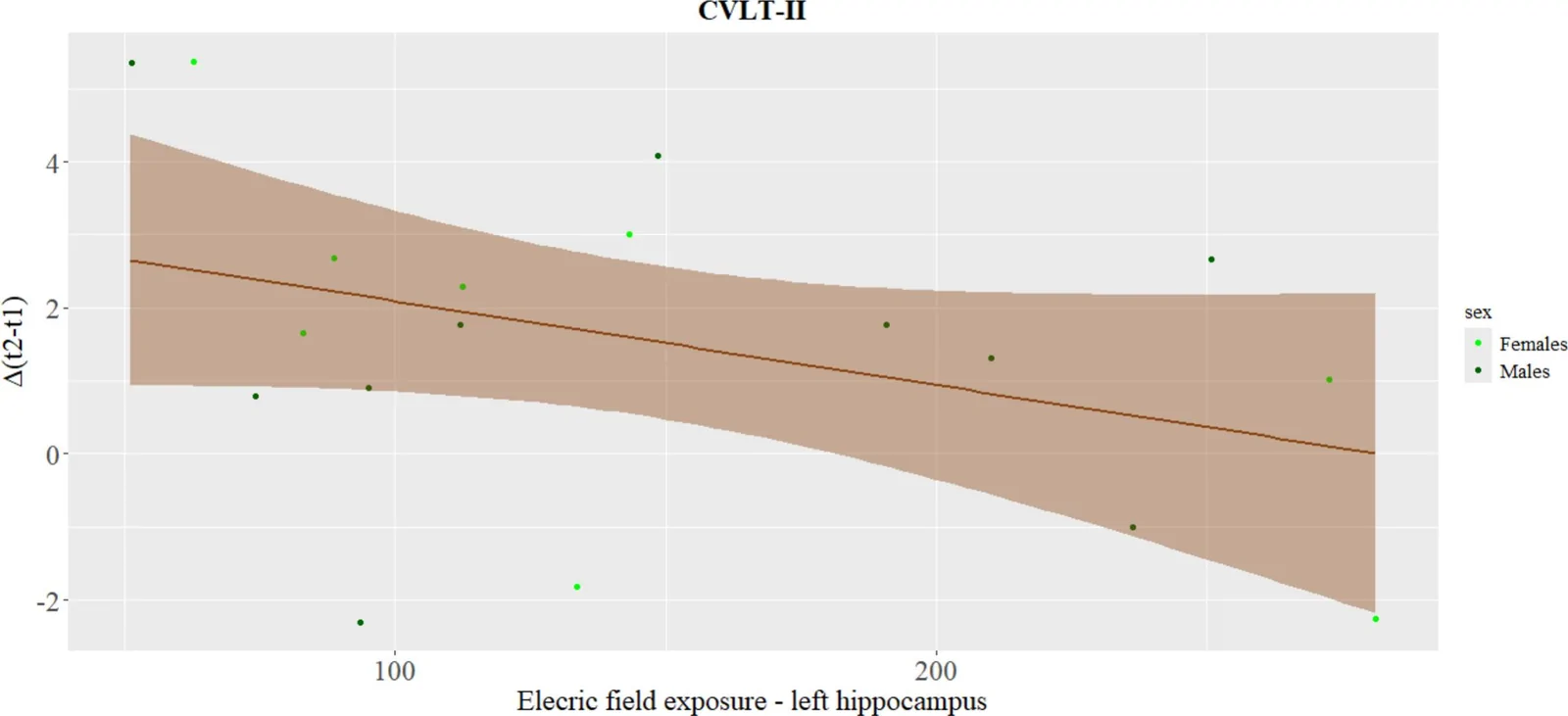
CVLT-II Δ scores associated with EF exposure in the left hippocampus. Relationship between EF exposure in the left hippocampus and change in CVLT-II performance. Positive Δ values (>0) are indicative of an improvement in performance on CVLT-II. Regression line represents adjusted linear regression model controlling for age, sex, ΔMADRS, and IQ. Shaded areas indicate 95% confidence intervals. Note: The EF values here differ from earlier figures because this model tested EF exposure (cumulative measure) rather than average EF strength. Abbreviations: CVLT-II = California Verbal Learning Test-Second Edition.

## 4 Discussion

The present study investigated the impact of EF strength on hippocampal volume and cognitive outcomes in patients affected by severe or TRD who underwent ECT, and we hypothesized that sex would influence these associations. Supporting this, female patients exhibited higher EF strengths in both hippocampi, which correlated with smaller intracranial volumes and greater left hippocampal volume changes after ECT. In addition, we found an effect of age, with older patients showing lower EF values. Patients, overall, had a reduction in depressive symptoms severity and no decline or sex-differences in cognitive performance. Greater hippocampal volume increase and higher EF strength in the left hippocampus were associated with smaller improvement in verbal memory performance and lower consistency in autobiographical memory. The association between left hippocampal volume change and verbal memory performance differed by sex, with volume increase correlating with greater improvement in performance in males but smaller in females.

As previously shown in Mossige (2021), age was negatively correlated with EF strength. In line with previous research (Deng et al., 2015), we suggest that the negative association between age and hippocampal EF strength may reflect age-related anatomical changes that influence current flow through the head. Potential contributors include changes in skull thickness and conductivity, brain volume, and cerebrospinal fluid spaces. Although the initial ECT-dose was based on age, we argue that the age-based dosing method inadequately accounts for individual variability in anatomy and body composition, potentially leading to unequal treatment effects. The present findings highlight the influence of individual anatomical differences on EF exposure during ECT. Female patients tended to have smaller intracranial volumes compared to males, which was functionally relevant. Intracranial volume was negatively correlated with EF strength in both the left and right hippocampus. This suggests that a smaller head size may lead to stronger EF strength under standard stimulation parameters. Importantly, female patients also showed significantly greater volume increases in the left hippocampus.

In line with previous findings (Argyelan et al., 2019), we observed a positive association between EF strength and hippocampal volume change, supporting the hypothesis that the EF plays a central role in ECT-induced neuroplasticity. Although the patient sample analyzed in the present study is a subset of the larger GEMRIC dataset used in Argyelan et al. (2019) (Argyelan et al., 2019), our results supports this relationship and extend it by incorporating individual-level variables such as sex and age.

There was no evidence of cognitive side effects after ECT when assessed at mean 16.5 days after the final ECT session, consistent across both females and males, and in line with previous studies (Landry et al., 2021; Anderson et al., 2021; Biedermann et al., 2016; Bodnar et al., 2016), although transient cognitive impairments earlier in the treatment course cannot be excluded (Landry et al., 2021; Semkovska and McLoughlin, 2010). It is also important to emphasize that the absence of cognitive side effects at the group level does not preclude the presence of individual differences. Patients vary substantially in anatomical characteristics, which in turn influence the distribution and strength of the induced EF in the brain. Anatomical factors discussed above all modulate EF strength and may contribute to differential vulnerability to cognitive side effects, influencing critical brain areas like the hippocampus. Indeed, higher EF strength in the left hippocampus significantly predicted less improvement in verbal memory performance (delayed recall of a word list), from baseline to post-treatment. A similar trend was observed for the right hippocampus, though it did not reach statistical significance. To our knowledge, this is the first study to report an association between EF and verbal memory performance following ECT.

Previous studies primarily focused on verbal fluency and found that higher EF strength was consistently associated with decline in verbal fluency measures, particularly in language-related regions of the left hemisphere (Loef et al., 2025), the right hippocampus (Abbott et al., 2024), and broader cognitive-impairment networks (Qi et al., 2022; Miller et al., 2023). Sample characteristics such as size, age, timing of assessments, and medication may have influenced the lack of a significant relationship between EF strength and verbal fluency in our study.

Given the hippocampus's central role in memory, it remains plausible that verbal memory may be particularly vulnerable to higher EF exposure. Supporting this, we also found that greater hippocampal volume changes after ECT predicted smaller improvement in performance on CVLT-II and lower AMI-SF consistency scores. These results align with previous studies reporting that hippocampal enlargement co-occurred with a decrease in cognitive functioning (Argyelan et al., 2021; Gbyl et al., 2021; Van Oostrom et al., 2018), although some have reported a positive (Leaver et al., 2021) or a null effect (Abbott et al., 2024). In sum, these findings raise the question of wheter ECT-induced hippocampal volume increases may, in some individuals, come at a cognitive cost in line with studies suggesting that neurogenesis impacts established hippocampus-dependent memories (Akers et al., 2014).

We found no significant sex differences in cognitive performance. However, given the sex differences we found in EF strength and hippocampal volume changes, we examined whether the association between those measures and cognitive outcomes differed by sex. While sex did not significantly interact with EF in relation to cognitive scores, possibly because anatomical sex-related factors (e.g., skull thickness, head diameter, and brain volume) already influence EF strength (Peterchev et al., 2010), we did observe significant moderation effects when examining hippocampal volume change, however, these results were quite heterogenous and, in the case of AMI-SF, results were driven by outliers. Moreover, the sex-based dose adjustment (5–10% increase of charge for male and decreased for female patients) to some degree affected EF exposure without changing EF strength in our fixed-current RUL ECT. This may have decreased the sex differences in cognition.

Although our findings are preliminary and based on a limited sample, they raise the question of whether future clinical practice should consider individual anatomical and demographic characteristics when determining ECT dosing, discussed below.

### 4.1 Clinical implications

While the association between EF exposure and cognitive outcomes adds to the understanding of ECT's neurobiological effects, the clinical utility of these insights is still emerging. Clinicians are already aware that some patients exhibit heightened vulnerability to cognitive side effects, and registry studies have shown that young female patients, in particular, frequently report subjective memory complaints following ECT (Brus et al., 2017). Our preliminary findings suggest that sex differences in EF strength and hippocampal volume change are largely anatomically driven, with smaller intracranial volume in females contributing to higher EF strength. However, sex did not moderate the EF-cognition relationship directly, indicating that anatomical variability, rather than sex per se, may be the more relevant factor in predicting cognitive outcomes.

This raises important questions about how to individualize treatment. Some ECT devices, such as the Thymatron, operate with fixed current amplitudes, which strictly determine peak EF strength. This presents a practical limitation when considering how to tailor stimulation to individual anatomy. While modulation of total charge (through adjustments in pulse number and duration) can influence the cumulative impact of neural activation, it does not alter the peak EF magnitude. Thus, adapting treatment to minimize cognitive side effects without compromising efficacy remains a complex challenge. Notably, Deng et al. (2022), using a Mecta device with variable pulse amplitudes, identified a maximal right hippocampal electric field (EF) of 112.5 V/m as a potential threshold for cognitive safety (Deng et al., 2022). They proposed that “*E*-field-informed ECT”, which individualizes current amplitude based on both the anatomical location (e.g., hippocampus) and the optimal EF strength (e.g., 112.5 V/m), may offer a promising path toward personalized treatment. Although our study was conducted using a Thymatron device with fixed current amplitude, our findings are among the first to empirically link EF exposure to hippocampal volume changes and cognitive performance. Taken together with the prior work (Deng et al., 2022; Abbott et al., 2024), results may represent an important step toward the development of more individualized and anatomically informed ECT protocols. Until such approaches are validated and implemented, clinicians should remain vigilant. When patients experience more pronounced memory difficulties than expected, it is essential to pause and consider adjustments to the treatment protocol or alternative interventions. These findings underscore the importance of ongoing cognitive monitoring and individualized care in ECT practice.

### 4.2 Limitations

The relatively small sample size limits statistical power and generalizability and increases sensitivity to outliers. Further, corrections for multiple testing were not applied due to the exploratory nature and the limited sample size, which increases the risk of false positives. However, we conducted *post-hoc* sensitivity analyses to assess the influence of outliers and made these effects explicit in the results. We did not exclude outliers to preserve the validity of our clinical sample and to ensure the inclusion of potential individuals experiencing side effects. Additionally, the timing of post-treatment cognitive assessments varied considerably (range: 1–53 days) which could have contributed to variability in the outcome measures. Further, we lack data on individual-level factors that may influence the cognitive outcomes, such as psychiatric comorbidities and premorbid cognitive status. Due to limited sample size, we did not include available data on medication use and pulse width. These factors may have contributed to variability in EF exposure and cognitive response. Finally, although the EF exposure variable was introduced to approximate cumulative stimulation effects, it should be considered a proxy measure rather than a direct quantification. ROAST simulations are based on a single time point and do not capture dynamic changes within and across sessions. While EF exposure improves physiological relevance by incorporating both field strength and total stimulation time, it remains an indirect estimate of cumulative neural impact and should be validated in future studies.

## 5 Conclusion

Higher EF strength correlated with greater hippocampal volume increase in the left hemisphere, and higher EF exposure in the left hippocampus was associated with delayed verbal memory performance. Although female patients exhibited higher EF strengths and larger hippocampal volume increases, likely reflecting anatomical factors, these differences did not translate into consistent effects on cognitive outcomes. The observed individual variability in EF exposure and cognitive response highlights the need for further research into personalized ECT approaches. Future studies should aim to replicate these findings in larger samples, and investigate whether adjusting stimulation parameters based on individual brain anatomy could optimize the balance between therapeutic efficacy and cognitive side effects, particularly in individuals who may be more vulnerable to cognitive adverse effects.

## CRediT authorship contribution statement

**Margherita Biondi:** Writing – review & editing, Writing – original draft, Formal analysis. **Eivind Haga Ronold:** Writing – review & editing, Supervision, Formal analysis. **Ingrid Mossige:** Writing – review & editing, Methodology, Investigation, Data curation. **Leif Oltedal:** Writing – review & editing, Project administration, Methodology, Conceptualization. **Olga Therese Ousdal:** Writing – review & editing, Supervision, Methodology. **Christopher C. Abbott:** Writing – review & editing, Supervision, Methodology. **Ketil Joachim Ødegaard:** Writing – review & editing, Methodology, Funding acquisition, Conceptualization. **Renate Grüner:** Writing – review & editing, Supervision, Methodology, Conceptualization. **Ute Kessler:** Writing – review & editing, Supervision, Project administration, Investigation, Data curation, Conceptualization.

## Ethics declaration

Written informed consent to take part in the study and to publish the article has been obtained from all participants or their legal representatives. The privacy rights of participants have been observed.

This study was performed in compliance with relevant laws, regulatory frameworks and guidelines where the research took place.

This study was approved by the Regional ethical committee Sør øst.

(Approval No. 2013/1032 ECT and neuroradiology)

## Declaration of competing interest

The authors declare that they have no known competing financial interests or personal relationships that could have appeared to influence the work reported in this paper.

## Data Availability

Data will be made available on request.
