# Supplementary material for: Electric field strength, hippocampal volume change, and cognitive outcomes following electroconvulsive therapy: the role of sex and anatomy
Source: Neuroimage Clin. 2026 Oct 9;52:104078. doi: 10.1016/j.nicl.2026.104078 (PMC13694425; doi:10.1016/j.nicl.2026.104078)
Supplement: Supplementary file 1 [file mmc1.docx]

**SUPPLEMENTARY MATERIALS**

**Electric Field Strength, Hippocampal Volume Change, and Cognitive Outcomes Following ECT: The Role of Sex and Anatomy**

Margherita Biondi ^a^, Eivind Haga Ronold ^b,c^, Ingrid Mossige ^d,e^, Leif Oltedal ^c,f^, Olga Therese Ousdal ^c,g^, Christopher C Abbott ^h^, Ketil Joachim Ødegaard ^f,i^, Renate Grüner ^d^, Ute Kessler ^c,f,i*^

^a^ Padova Neuroscience Center, University of Padova, Via Giuseppe Orus, 2, 35131 Padova, Italy

^b^ Department of Clinical and Biological Psychology, University of Bergen, Christies gate 12, 5015 Bergen, Norway

^c^ Mohn Medical Imaging and Visualization Centre, Department of Radiology, Haukeland University Hospital, Haukelandsveien 22, 5009 Bergen, Norway

^d^ Department of Physics and Technology, University of Bergen, Allégaten 55, 5007 Bergen, Norway

^e^ Department of Physics and Computational Radiology, Division of Radiology and Nuclear Medicine, Oslo University Hospital, Sognsvannveien 21, 0372 Oslo, Norway

^f^ Department of Clinical Medicine, University of Bergen, Haukelandsveien 28, 5009 Bergen, Norway

^g^ Department of Biomedicine, Faculty of Medicine, University of Bergen, Jonas Lies vei 91, 5009 Bergen, Norway

^h^ Department of Psychiatry, University of New Mexico, 2600 Marble Ave, Albuquerque, NM 87106, USA

^i^ Department of Psychiatry, Haukeland University Hospital, Sandviksleitet 1, 5036 Bergen, Norway

*Correspondence: Ute Kessler, Department of Psychiatry, Haukeland University Hospital, Sandviksleitet 1, 5036 Bergen, Norway, ute.kessler@helse-bergen.no

**SUPPLEMENTARY METHODS**

**M1) MRI acquisition: full parameters**

Systems and coils: The majority of patients were scanned on a 3T GE Discovery MR750 system with a 32-channel head coil. Eight patients were scanned using a 3T GE Signa HDxt system with an 8-channel head coil before the hospital MR scanner got upgraded. Each participant underwent both MRI scans using the same system to ensure consistency.

Timing: MRI‑1: 1–2 h prior to first ECT; MRI‑2: 7–14 days after the final session; typical start time 08:00–09:00.

Sequence parameters: The T1-weighted images were acquired using a fast spoiled gradient echo (FSPGR) pulse sequence (TR/TE=2.9/6.7 ms, TI=600 ms, α=8°, FOV=25.6 cm, voxel size=1.0×1.0×1.0 mm3, acquisition time=10:32 min).

**M2) Image analysis and EF simulation: processing details**

Image preprocessing included correction for scanner-specific gradient field nonlinearities [1] and intensity for non-uniformity in the B0 field. Segmentation of hippocampal volumes were performed using FreeSurfer version 7.1.1, applying the longitudinal processing pipeline [2]. This involved three steps: (1) cross-sectional processing of pre-treatment (MRI 1) and post-treatment (MRI 2) T1-weighted images using recon-all; (2) creation of an unbiased within-subject template (base) from both time points for each subject; and (3) longitudinal processing using this base (FreeSurfer, n.d.). To quantify the percentage change in hippocampal volume (left and right), the difference between post-treatment and pre-treatment volumes was divided by the pre-treatment volume. Estimated total intracranial volume was derived from T1-weighted MRI scans using FreeSurfer’s automated segmentation pipeline. Details on image processing and analysis can be found in Mossige (2021) [3].

The EF generated in the brain during ECT was simulated using ROAST version 3.0 [4] with the pre-treatment T1-weighted images as input. Simulations used a 900 mA current, disc electrodes (25 mm radius), and right unilateral placement (C2 and FFT8, 10–05 EEG system). ROAST segmented the head into air cavities and five different tissue compartments (white matter, grey matter, cerebrospinal fluid, bone, and scalp) using SPM12. Conductivity was assigned to each compartment [4]. Furthermore, a tetrahedral Finite Element Model (FEM) mesh was generated from the segmented MRI and solved for voltage and the EF. The resulting EF strength maps were cropped and reoriented to FreeSurfer space. Mean EF strengths in the left and right hippocampus were calculated after excluding the top and bottom 1% of values to reduce outlier influence. ROAST-derived EF modeling is displayed in Figure S1 (see SUPPLEMENTARY FIGURES section). For further methodological details, see Mossige (2021) [3].

Since ROAST simulates EF based on a single time point (i.e., the injected current of 900 mA), without accounting for cumulative ECT stimulation, the EF exposure was introduced as a variable that better reflect the EF over the time course of treatment. EF exposure was defined as the product of the average EF strength within a brain region and the total exposure time, calculated as the cumulative duration (in seconds) of all pulse widths across ECT sessions [5].

**M3) Cognitive assessment: tests description**

Cognitive assessments were carried out by a trained test technician, on average 4 days (range, 2 – 6) before the first ECT and on average 16.5 days (range, 1 – 53) after the last ECT session with the following tests:

-California Verbal Learning Test-Second Edition (CVLT-II): Assessed verbal long-term memory using delayed free recall scores [6]. Higher scores indicate better performance.

-Rey Osterrieth Complex Figure Test (REY): Evaluated visual long-term memory by scoring the reproduction of a complex figure after a 30-minute delay [7]. Raw scores based on parts of the figure correctly remembered were used, and higher scores are indicative of better performance.

-Verbal Fluency Test (VF): Measured verbal fluency using the Letter and Category Fluency subtests from the D-KEFS battery [8]. A composite score was calculated as the average of total words generated across both conditions, and high score indicates better performance.

-Autobiographical Memory Interview-Short Form (AMI-SF): Assessed retrograde autobiographical memory consistency across two time points [9]. Scores reflect the percentage of consistent responses post-ECT with high score indicating better performance.

-Everyday Memory Questionnaire (EMQ): A 28-item self-report measure of subjective memory complaints in daily life [10]. Higher scores are indicative of more subjective complaints.

-Wechsler Abbreviated Scale of Intelligence (WASI): Provided an estimate of general intellectual functioning based on Verbal and Performance intelligence quotient (IQ) subtests [11].

Results from these assessments have been previously reported [12, 13].

**SUPPLEMENTARY RESULTS**

Below are provided detailed outputs from: a) indipendent t-tests comparing clinical and cognitive scores between t1 and t2 on CVLT-II, REY, VF, and EMQ (Table S1); b) indipendent t-tests (or Mann-Whitney U tests for non-normal data) comparing Δ cognitive scores (t2-t1) and post-ECT values (for AMI-SF) between females and males (Table S2); c) adjusted and interaction linear models examining associations between hippocampal volume change and Δ cognitive scores (t2-t1) (Table S3 and Table S4); d) adjusted and interaction linear models examining associations between EF exposure and Δ cognitive scores (t2-t1) (Table S5 and Table S6). All models were adjusted for sex, age, clinical effect (i.e., change in MADRS score (t2−t1)), and IQ (for CVLT-II, REY, VF). Interaction terms were included to test sex-specific effects. Reported *p*-values are uncorrected for multiple comparisons; therefore, findings should be interpreted as exploratory.

**a) Clinical and cognitive assessment**

Indipendent t-tests compared clinical and cognitive scores between t1 and t2 (Table S1). ECT patients had reduced MADRS scores after treatment (*t*=11.01, *p*<0.001) (Table S1). There was no significant change in performance on CVLT-II, REY, and VF assessments nor in subjective memory complaints on the EMQ (Table S1). AMI-SF scores are also reported in Table S1.

**Table S1. Clinical and cognitive assessment**

| **Test** | **N** | **t1 (pre-ECT)** | | | **t2 (post-ECT)** | | | **Mean Δ**  **(t2−t1) **** | ***t*-value**  **t1 vs t2** | ***p*-value** | |
| --- | --- | --- | --- | --- | --- | --- | --- | --- | --- | --- | --- |
| **MADRS*** | 29  (f=15/m=14) | 33.59 | ± | 4.99 | 14.48 | ± | 7.71 | -19.11  (ES= 3.8) | 11.01 | < 0.001 | |
| **CVLT-II** | 18  (f=8/m=10) | 9. | ± | 3.92 | 11 | ± | 3.84 | +1.56  (ES= 0.4) | -1.17 | 0.25 | |
| **REY** | 18  (f=8/m=10) | 13.08 | ± | 5.71 | 16.58 | ± | 5.91 | +3.58  (ES= 0.6) | -1.76 | 0.09 | |
| **VF** | 17  (f=8/m=9) | 39.21 | ± | 7.94 | 40.1 | ± | 10.25 | +0.89  (ES= 0.1) | -0.26 | 0.79 | |
| **EMQ*** | 27  (f=13/m=14) | 118. | ± | 32.11 | 107.93 | ± | 29.73 | -10.  (ES= 0.3) | 1.21 | 0.23 | |
| **Only post-ECT test** | | | | |  | | | | | |  |
| **AMI-SF** | 20  (f=8/m=12) | **Consistency score** | | |  | | | | | | |
|  |  | 85.49 (%) | | |  |  |  |  |  |  |  |

* Lower MADRS scores are indicative of a better condition, and lower EMQ scores are indicative of a better performance.

** Positive Δ values (>0) are indicative of an improvement in performance. The opposite applies in the case of MADRS and EMQ.

Abbreviations: AMI-SF= Autobiographical Memory Interview-Short Form, CVLT-II= California Verbal Learning Test-Second Edition, ECT= Electroconvulsive Therapy, EMQ= Everyday Memory Questionnaire, ES= Effect Size, MADRS= Montgomery and Åsberg Depression Rating Scale, REY= Rey Osterrich Complex Figure, VF= Verbal Fluency.

**b) Sex differences in cognitive performance**

Independent t-tests (or Mann-Whitney U tests for non-normal data) compared Δ cognitive scores (t2-t1) and post-ECT values (for AMI-SF) between females and males (Table S2). No significant sex differences were observed across cognitive domains.

**Table S2. Sex differences in cognitive performance**

|  | **Mean Δ (t2−t1) **** | | | | ***t/W* statistic** | ***p*-value** |
| --- | --- | --- | --- | --- | --- | --- |
|  | **Females** | | **Males** | |  |  |
| **CVLT-II** | Pre-ECT score= 9.5 | +1.5 | Pre-ECT score= 9.4 | +1.6 | *t*=-0.09 | 0.93 |
|  | Post-ECT score= 11 |  | Post-ECT score= 11 |  |  |  |
| **REY** | Pre-ECT score= 14.37 | +2.31 | Pre-ECT score= 12.05 | +4.45 | *t*=-0.81 | 0.43 |
|  | Post-ECT score= 16.68 |  | Post-ECT score= 16.5 |  |  |  |
| **VF** | Pre-ECT score= 38.68 | +2.94 | Pre-ECT score= 39.66 | -1 | *t*=1.47 | 0.16 |
|  | Post-ECT score= 41.62 |  | Post-ECT score= 38.66 |  |  |  |
| **EMQ*** | Pre-ECT score= 126.23 | -10.38 | Pre-ECT score= 111 | -10.43 | *t*=-0.003 | 0.99 |
|  | Post-ECT score= 115.85 |  | Post-ECT score= 100.57 |  |  |  |
|  | **Only post-ECT test** | | | |  | |
|  | **Females** | | **Males** | |  |  |
| **AMI-SF** | 87.79% | | 83.95% | | *W*=58.5 | 0.44 |

* Lower EMQ scores are indicative of a better performance.

** Positive Δ values (>0) are indicative of an improvement in performance. The opposite applies in the case of EMQ.

Abbreviations: AMI-SF= Autobiographical Memory Interview-Short Form, CVLT-II= California Verbal Learning Test-Second Edition, ECT= Electroconvulsive Therapy, EMQ= Everyday Memory Questionnaire, REY= Rey Osterrich Complex Figure, VF= Verbal Fluency.

**c) Change in cognitive performance and the association with hippocampal volume change**

The full adjusted (Table S3) and interaction linear models (Table S4) for our analyses are provided below. Adjusted models and interaction models tested associations between hippocampal volume change (left/right) and Δ cognitive scores (t2-t1), correcting for sex, age, and clinical effect (i.e., change in MADRS score (t2−t1)) and IQ (only for CVLT-II, REY, VF).

Significant findings were limited to CVLT-II (left hippocampus): main effect (*p*=0.04) and interaction with sex (*p*=0.05), and AMI-SF (left hippocampus): main effect (*p*=0.01) and interaction with sex (*p*=0.04) (Table S4). All other associations were non-significant.

**Table S3. Change in cognitive performance and the association with hippocampal volume change: adjusted linear models**

| **Cognitive Test** | **Hemisphere** | ***b***  **(Hippocampal volume change)** | **95% CI** | ***t*-value** | ***p*-value** |
| --- | --- | --- | --- | --- | --- |
| **Δ CVLT-II** | left | -0.29 | [-1.08,0.5] | -0.79 | 0.44 |
|  | right | -0.23 | [-0.86,0.39] | -0.8 | 0.44 |
| **Δ REY** | left | 0.27 | [-1.77,2.32] | 0.29 | 0.77 |
|  | right | 0.09 | [-1.52,1.71] | 0.13 | 0.9 |
| **Δ VF** | left | 0.24 | [-1.46,1.95] | 0.32 | 0.76 |
|  | right | -0.08 | [-1.58,1.41] | -0.12 | 0.91 |
| **Δ EMQ** | left | 2.08 | [-6.06,10.24] | 0.53 | 0.6 |
|  | right | 1.69 | [-4.75,8.15] | 0.54 | 0.59 |
| **AMI-SF** | left | -2.54 | [-5.9,0.82] | -1.61 | 0.13 |
|  | right | -2.16 | [-4.85,0.53] | -1.71 | 0.11 |

Abbreviations: AMI-SF= Autobiographical Memory Interview-Short Form, CI= Confidence Interval, CVLT-II= California Verbal Learning Test-Second Edition, EMQ= Everyday Memory Questionnaire, REY= Rey Osterrich Complex Figure, VF= Verbal Fluency.

**Table S4. Change in cognitive performance and the association with hippocampal volume change: interaction linear models with sex**

| **Cognitive Test** | **Hemisphere** | ***b* (Hippocampal volume change)** | **95% CI** | ***t*-value** | ***p*-value** | ***b* Interaction (Hippocampal volume change x sex)** | **95% CI** | **Interaction *t*-value** | **Interaction *p*-value** |
| --- | --- | --- | --- | --- | --- | --- | --- | --- | --- |
| **Δ CVLT-II** | **left** | **-2.21** | **[-4.3,-0.12]** | **-2.33** | **0.04** | **1.37** | **[-0.03,2.76]** | **2.15** | **0.05** |
|  | right | -1.62 | [-3.49,0.25] | -1.91 | 0.08 | 0.96 | [-0.27,2.19] | 1.72 | 0.11 |
| **Δ REY** | left | -1.9 | [-8.14,4.34] | -0.67 | 0.52 | 1.55 | [-2.63,5.72] | 0.81 | 0.43 |
|  | right | -1.72 | [-7.02,3.58] | -0.71 | 0.49 | 1.26 | [-2.24,4.75] | 0.79 | 0.44 |
| **Δ VF** | left | 3.78 | [-1.2,8.77] | 1.72 | 0.12 | -2.25 | [-5.5,1] | -1.56 | 0.15 |
|  | right | 2.84 | [-2.04,7.72] | 1.32 | 0.22 | -1.61 | [-4.75,1.53] | -1.16 | 0.27 |
| **Δ EMQ** | left | -13.05 | [-36.24,10.15] | -1.17 | 0.25 | 10.72 | [-4.71,26.16] | 1.44 | 0.16 |
|  | right | -0.55 | [-20.92,19.82] | -0.06 | 0.96 | 1.64 | [-12.39,15.67] | 0.24 | 0.81 |
| **AMI-SF** | **left** | **-4.87** | **[-8.56,-1.17]** | **-2.83** | **0.01** | **5.31** | **[0.35,10.26]** | **2.29** | **0.04** |
|  | right | -3.03 | [-7.06,1] | -1.61 | 0.13 | 1.69 | [-4.03,7.41] | 0.63 | 0.54 |

Abbreviations: AMI-SF= Autobiographical Memory Interview-Short Form, CI= Confidence Interval, CVLT-II= California Verbal Learning Test-Second Edition, EMQ= Everyday Memory Questionnaire, REY= Rey Osterrich Complex Figure, VF= Verbal Fluency.

**d) Change in cognitive performance and the association with EF exposure**

The full adjusted (Table S5) and interaction linear models are provided below (Table S6). Adjusted models and interaction models tested the association between EF exposure (left/right hippocampus) and Δ cognitive scores (t2-t1), correcting for sex, age, and clinical effect (i.e., change in MADRS score (t2−t1)) and IQ (only for CVLT-II, REY, VF). Significant findings were limited to CVLT-II (left hippocampus): main effect (*p*=0.05) (Table S6). All other associations were non-significant.

**Table S5. Change in cognitive performance and the association with EF exposure: adjusted linear models**

| **Cognitive Test** | **Hemisphere** | ***b* (EF exposure)** | **95% CI** | ***t*-value** | ***p*-value** |
| --- | --- | --- | --- | --- | --- |
| **Δ CVLT-II** | **left** | **-0.02** | **[-0.04,1.72]** | **-2.16** | **0.05** |
|  | right | -0.01 | [-0.02,0] | -2.03 | 0.06 |
| **Δ REY** | left | -0.04 | [-0.05,0.02] | -1.81 | 0.09 |
|  | right | -0.02 | [-0.06,0] | -1.81 | 0.1 |
| **Δ VF** | left | 0.01 | [-0.03,0.06] | 0.57 | 0.58 |
|  | right | 0.01 | [-0.02,0.04] | 0.86 | 0.41 |
| **Δ EMQ** | left | 0.001 | [-0.24,0.24] | 0.01 | 0.99 |
|  | right | 0.03 | [-0.12,0.18] | 0.41 | 0.69 |
| **AMI-SF** | left | -0.03 | [-0.12,0.07] | -0.57 | 0.58 |
|  | right | -0.01 | [-0.07,0.04] | -0.54 | 0.59 |

Abbreviations: AMI-SF= Autobiographical Memory Interview-Short Form, CI= Confidence Interval, CVLT-II= California Verbal Learning Test-Second Edition, EF= Electric Field, EMQ= Everyday Memory Questionnaire, REY= Rey Osterrich Complex Figure, VF= Verbal Fluency.

**Table S6. Change in cognitive performance and the association with EF exposure: interaction linear models with sex**

| **Cognitive Test** | **Hemisphere** | ***b* (EF exposure)** | **95% CI** | ***t*-value** | ***p*-value** | ***b* Interaction (EF exposure x sex)** | **95% CI** | **Interaction *t*-value** | **Interaction *p*-value** |
| --- | --- | --- | --- | --- | --- | --- | --- | --- | --- |
| **Δ CVLT-II** | left | -0.02 | [-0.08,0.02] | -1.1 | 0.29 | 0.005 | [-0.03,0.04] | 0.32 | 0.75 |
|  | right | -0.01 | [-0.05,0.02] | -1 | 0.31 | 0.003 | [-0.02,0.02] | 0.31 | 0.76 |
| **Δ REY** | left | -0.06 | [-0.19,0.07] | -1.07 | 0.31 | 0.02 | [-0.07,0.11] | 0.42 | 0.68 |
|  | right | -0.04 | [-0.12,0.04] | -1.14 | 0.28 | 0.01 | [-0.04,0.07] | 0.49 | 0.63 |
| **Δ VF** | left | 0.007 | [-0.12,0.13] | 0.12 | 0.91 | 0.004 | [-0.09,0.09] | 0.09 | 0.92 |
|  | right | 0.001 | [-0.08,0.08] | 0.03 | 0.98 | 0.007 | [-0.05,0.06] | 0.3 | 0.77 |
| **Δ EMQ** | left | -0.14 | [-0.84,0.55] | -0.43 | 0.67 | 0.09 | [-0.35,0.54] | 0.46 | 0.65 |
|  | right | -0.11 | [-0.52,0.3] | -0.56 | 0.58 | 0.09 | [-0.17,0.36] | 0.76 | 0.45 |
| **AMI-SF** | left | 0.02 | [-0.12,0.17] | 0.38 | 0.71 | -0.09 | [-0.26,0.08] | -1.07 | 0.3 |
|  | right | 0.01 | [-0.07,0.09] | 0.38 | 0.71 | -0.05 | [-0.15,0.05] | -1.11 | 0.29 |

Abbreviations: AMI-SF= Autobiographical Memory Interview-Short Form, CI= Confidence Interval, CVLT-II= California Verbal Learning Test-Second Edition, EF= Electric Field, EMQ= Everyday Memory Questionnaire, REY= Rey Osterrich Complex Figure, VF= Verbal Fluency

**SUPPLEMENTARY FIGURES**

**Figure S1. ROAST-derived EF modeling**

Axial, coronal and sagittal view of the simulated EF distribution across the whole head in a random participant (MRI 1). The two electrodes are shown in white and light orange. The colourmap was scaled to see contrast in the brain. EF strength values above 300 V/m are colored as 300 V/m. Previously presented in Mossige (2021) [3].

**Figure S2. Hippocampal volume change by number of ECT sessions**

Scatterplots showing percentage volume change in the left and right hippocampus as a function of the total number of ECT sessions. The number of ECT sessions was positively associated with right hippocampal volume change, whereas the association with left hippocampal volume change was not significant. Previously presented in Mossige (2021) [3].

**SUPPLEMENTARY REFERENCES**

[1]	Jovicich J, Czanner S, Greve D, Haley E, Van Der Kouwe A, Gollub R, et al. Reliability in multi-site structural MRI studies: Effects of gradient non-linearity correction on phantom and human data. NeuroImage. 2006; 30: 436–443. https://doi.org/10.1016/j.neuroimage.2005.09.046.

[2]	Reuter M, Schmansky NJ, Rosas HD, Fischl B. Within-subject template estimation for unbiased longitudinal image analysis. NeuroImage. 2012; 61: 1402–1418. https://doi.org/10.1016/j.neuroimage.2012.02.084.

[3]	Mossige I. *Volume Increase of the Hippocampus after Electroconvulsive Therapy - The Role of the Electric Field and the Individual Differences due to Body Mass Index, Sex and Age*, https://hdl.handle.net/11250/2763471 (2021).

[4]	Huang Y, Datta A, Bikson M, Parra LC. Realistic volumetric-approach to simulate transcranial electric stimulation—ROAST—a fully automated open-source pipeline. J Neural Eng. 2019; 16: 056006. https://doi.org/10.1088/1741-2552/ab208d.

[5]	Yang C, Wu H, Wu N, Weng S, Wang D, Wang W, et al. Intracranial electric field dosage and clinical response to electroconvulsive therapy in depressed patients. J Psychiatr Res. 2025; 190: 417–424. https://doi.org/10.1016/j.jpsychires.2025.08.021.

[6]	Woods S, Delis D, Scott J, Kramer J, Holdnack J. The California Verbal Learning Test – second edition: Test-retest reliability, practice effects, and reliable change indices for the standard and alternate forms. Arch Clin Neuropsychol. 2006; 21: 413–420. https://doi.org/10.1016/j.acn.2006.06.002.

[7]	Shin M-S, Park S-Y, Park S-R, Seol S-H, Kwon JS. Clinical and empirical applications of the Rey–Osterrieth Complex Figure Test. Nat Protoc. 2006; 1: 892–899. https://doi.org/10.1038/nprot.2006.115.

[8]	Delis DC, Kaplan E, Kramer JH. Delis-Kaplan Executive Function System. Epub ahead of print 8 October 2012. DOI: 10.1037/t15082-000.

[9]	McElhiney MC, Moody BJ, Sackeim HA. The Autobiographical Memory Interview Short Form: Manual for Administration and Scoring. *Department of Biological Psychiatry, New York State Psychiatric Institute.*, 2001.

[10]	Royle J, Lincoln NB. The Everyday Memory Questionnaire – revised: Development of a 13-item scale. Disabil Rehabil. 2008; 30: 114–121. https://doi.org/10.1080/09638280701223876.

[11]	Wechsler D. Wechsler Abbreviated Scale of Intelligence.

[12]	Blomberg MO, Semkovska M, Kessler U, Erchinger VJ, Oedegaard KJ, Oltedal L, et al. A Longitudinal Comparison Between Depressed Patients Receiving Electroconvulsive Therapy and Healthy Controls on Specific Memory Functions. Prim Care Companion CNS Disord. 22(3), 19m02547. https://doi.org/10.4088/PCC.19m02547.

[13]	Hammar Å, Ronold EH, Spurkeland MA, Ueland R, Kessler U, Oedegaard KJ, et al. Improvement of persistent impairments in executive functions and attention following electroconvulsive therapy in a case control longitudinal follow up study. BMC Psychiatry. 2024; 24: 832. https://doi.org/10.1186/s12888-024-06270-5.
